# Enhancement of Superconductivity in WP via Oxide-Assisted Chemical Vapor Transport

**DOI:** 10.3390/ma18194529

**Published:** 2025-09-29

**Authors:** Daniel J. Campbell, Wen-Chen Lin, John Collini, Yun Suk Eo, Yash Anand, Shanta Saha, David Graf, Peter Y. Zavalij, Johnpierre Paglione

**Affiliations:** 1 Maryland Quantum Materials Center, Department of Physics, University of Maryland, College Park, MD 20742, USAyanand@mit.edu (Y.A.);; 2 National High Magnetic Field Laboratory, 1800 East Paul Dirac Drive, Tallahassee, FL 32310, USA; 3 Department of Chemistry, University of Maryland, College Park, MD 20742, USA; 4 Canadian Institute for Advanced Research, Toronto, ON M5G 1Z8, Canada

**Keywords:** superconductivity, quantum materials

## Abstract

Tungsten monophosphide (WP) has been reported to superconduct below 0.8 K, and theoretical work has predicted an unconventional Cooper pairing mechanism. Here we present data for WP single crystals grown by means of chemical vapor transport (CVT) of ${\mathrm{WO}}_{3}$, P, and $I_{2}$. In comparison to synthesis using WP powder as a starting material, this technique results in samples with substantially decreased low-temperature scattering and favors a more three-dimensional morphology. We also find that the resistive superconducting transitions in these samples begin above 1 K. Variation in $T_{c}$ is often found in strongly correlated superconductors, and its presence in WP could be the result of influence from a competing order and/or a non-*s*-wave gap.

## 1. Introduction

Though first investigated half a century ago [1,2,3], MnP (or B31)-type transition metal pnictide binaries have been the subject of renewed attention in recent years. The various members show a wide variety of properties of interest in condensed matter physics: unusual spin density wave ordering [4], doping- and pressure-induced superconductivity [5,6,7,8], and topological band structure features [9,10,11]. Recently, superconductivity was found in WP at 0.8 K [12]. Despite the low reported $T_{c}$ and $H_{c2}$ (<20 mT), the superconducting state in WP attracts attention because it is predicted to be topologically nontrivial, like those observed in isostructural CrAs and MnP under pressure [10,13]. This is thought to be a result of nonsymmorphic crystallographic symmetries, which protect topologically nontrivial elements of the band structure [10]. The fact that superconductivity is present in WP without the need to apply pressure makes it the most appealing of the three materials for continued study and application.

Producing WP requires combining W, which has the highest melting point of any metal, with a very high vapor pressure element in P. It can therefore be challenging to find an appropriate temperature for crystal growth. Here, we sidestep the difficulty of a direct reaction between these two elements by using ${\mathrm{WO}}_{3}$ for single-crystal synthesis. Single crystals grown through chemical vapor transport of ${\mathrm{WO}}_{3}$, P, and $I_{2}$ (as the transport agent) are typically more three-dimensional than the long, thin needles reported in previous work [12,13]. These samples also have a lower residual resistivity, a sign of reduced low-temperature scattering. With this comes a large magnetoresistance (MR) that is linear in one orientation up to 20 T, another property that has been associated with topological band structure points in isostructural CrAs [9] and FeP [11]. Most notably of all, the onset of superconductivity in transport measurements comes at temperatures as high as 1.3 K in oxide-grown samples. A disorder-dependent $T_{c}$ is an indicator of unconventional superconductivity [14], and the unusual broadening of the transition at higher temperature that we see occurs in other unconventional superconductors as a result of competing orders [15,16,17]. The discovery via X-ray of multiple crystal modulation vectors could be related to this. The technique with which WP is grown has a clear influence on its properties. Here we outline our procedure and go through the measured properties, including those that are only now apparent with the obtained three-dimensional geometry.

## 2. Materials and Methods

Transport measurements were performed in a Quantum Design Physical Properties Measurement System, where temperatures below 1.8 K were achieved with either a ^3^He refrigerator or an adiabatic demagnetization refrigerator (ADR). The magnetic field was oscillated to zero to remove any trapped flux before measurement. High-magnetic-field measurements were taken with a 41.5 T resistive magnet and ^3^He system at the National High Magnetic Field Laboratory (NHMFL). Magnetic susceptibility was measured with a 7 T Quantum Design SQUID-VSM Magnetic Properties Measurement System. Powder X-ray diffraction (XRD) measurements were performed with a Rigaku Miniflex diffractometer using Cu $K_{\alpha }$ radiation. Single-crystal diffraction was performed with a Bruker Smart Apex II CCD diffractometer on two crystals at temperatures between 120 and 300 K. The integral intensity was corrected for absorption using SADABS software [18] using a multi-scan method. The resulting minimum and maximum transmissions are 0.033 and 0.082, respectively. The structure was solved with the ShelXT (Sheldrick, 2015a) [19] program and refined with the ShelXL [20] program and least-square minimization using the ShelX software package [20]. Single crystals were aligned using both the Miniflex and Laue photography and further elementally characterized with energy-dispersive X-ray spectroscopy (EDS).

Chemical vapor transport (CVT) has been used to grow high-quality crystals of practically all other phosphides in the MnP-type structure family [3,21,22]. For this technique, reactants are placed at one end of an evacuated quartz ampule with a temperature gradient along its length. This temperature gradient results in different solid–gas reaction rates at the two ends of the ampule and, if performed correctly, favors crystallization of the desired material at the ampule end that was originally empty. Iodine acts as a transport agent, whose high vapor pressure facilitates the evaporation and movement of the other materials down the length of the tube. The high vapor pressure of phosphorus means that many P-containing materials can be readily synthesized with this technique.

In the only reports on superconducting WP, crystals were grown via CVT with prereacted WP powder and iodine [12,13,23]. However, in the case of WP, this procedure has several possible pitfalls. Our work with FeP has shown that samples had a much lower residual resistivity in CVT growths starting from the elements compared to when prereacted FeP powder was used [11]. The residual resistivity is the baseline, temperature-independent value seen at the lowest temperatures and comes primarily from inherent impurity scattering. This means that lower values generally signify a smaller impurity concentration.

Another concern is the vastly different behavior of the two elements at high temperature. Red phosphorus (the least volatile form of the element and thus the one used for crystal growth) sublimes below 600 °C, while tungsten does not melt until 3400 °C and has one of the lowest vapor pressures of any element. It is difficult to find a middle point between these two for a reaction, especially given that, in practice, we are also limited by the fact that quartz ampules will soften or melt above about 1250 °C; there are, in fact, few examples of CVT involving pure W [24]. For that reason we took inspiration from an earlier work that used the oxides of heavy transition metals (W, Hf, Ta, and others) to produce single-crystal transition metal phosphides with CVT [25], which was employed in later work to produce WP as well as ${\mathrm{WP}}_{2}$ [26,27]. ${\mathrm{WO}}_{3}$ has a much lower melting point (1473 °C) than W; it is also thought that the release of oxygen at high temperature in combination with $I_{2}$ is beneficial to gas-phase transport [24,26]. Another group has reported using extremely high temperatures and pressures (3200 °C and 5 GPa) to achieve a congruent W-P melt [28] and produce large crystals. However, the need for significantly higher temperature and pressure makes such synthesis technically challenging.

**Figure 1:**
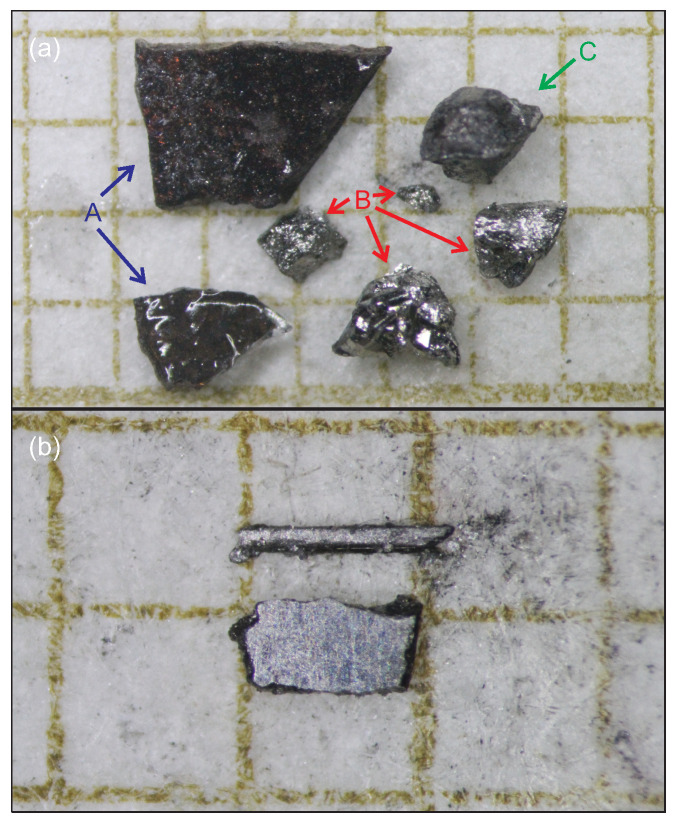
(**a**) The different materials that form at the cold end of a sealed quartz ampule when starting with ${\mathrm{WO}}_{3}$, P, and $I_{2}$ at the hot end. Pieces marked A are chunks of $W_{8}P_{4}O_{32}$, those marked B are WP (either single crystals or multiple intergrown crystals), and C is a piece of WP fused to a piece of $W_{8}P_{4}O_{32}$. (**b**) A comparison of a (top) needlelike and (bottom) platelike crystal of WP after initial polishing of only a single pair of opposing faces. The latter morphology was much more common in oxide-assisted growth. The grid paper in both pictures is composed of $1\times 1{\mathrm{mm}}^{2}$ squares.

For our CVT growths, ${\mathrm{WO}}_{3}$ (CERAC, 99.9% pure) and red P (Sigma Aldrich, >99.99% trace metals basis) powders were ground together in a 1:1 ratio and placed into a quartz ampule with additional $I_{2}$ (J.T. Baker, 99.9%, about $1\;{\mathrm{mg/cm}}^{3}$). The ampule was half the length of a single-zone tube furnace (about 15 cm) and oriented so that the reactants were initially in the middle of the furnace, at temperatures in the range 900– 1000 °C. The ampule end at the edge of the furnace was about 200 °C colder, and the growth was left for 10–14 days. Afterwards, it was found that some powder remained at the hot end, while a mixture of powder and crystalline material was at the cold end [Figure 1a]. XRD of the hot-end powder showed that it was pure WP, while that at the cold end was a mixture of WP powder and single crystals, which were black and shiny, with larger, red-tinged chunks of $W_{8}P_{4}O_{32}$ (equivalently, $W_{2}O_{4}$[${\mathrm{PO}}_{4}$]). Often the WP crystals were found fused to the $W_{8}P_{4}O_{32}$, but the combination could be polished to leave just the binary [Figure 1b, lower sample], with the absence of the other phase confirmed by XRD, EDS, and low-temperature measurements. The crystals produced in this growth are much more three-dimensional than the needles reported with WP powder-based growth [12,13], though a small number of needlelike crystals were found in oxide-based growths as well [Figure 1b, upper sample]. Subsequent attempts to use the WP powder that remained at the hot end for a new CVT growth with $I_{2}$ and a similar temperature and time profile to the oxide growth resulted in the powder staying in the hot region, without transporting to the cold ampule end. This is further evidence that oxygen is a critical part of the transport and crystal nucleation processes.

## 3. Results

### 3.1. Normal-State Properties

A 3D single crystal was chosen from one of the growths for detailed XRD measurements at 120 K. It was confirmed to be in the MnP-type Pnma orthorhombic structure with lattice parameters *a* = 5.7322(5) Å, *b* = 3.2487(3) Å, and *c* = 6.2246(5) Å, quite similar to those in previous reports at ambient temperature [12,25,29] in spite of possible thermal expansion effects. Further results from the structural refinement are given in Table 1. XRD was also performed on a crystal grown with the oxide method that had a more needlelike shape (such as the upper sample in Figure 1b) with very similar results. The X-ray measurements picked out a superstructure modulation vector of ($0\frac{1}{7}$ $\frac{1}{7}\;\frac{1}{7}$) in both samples, as well as additional vectors of ($0\;\frac{1}{2}\;\frac{1}{2}$), ($\frac{1}{2}\;0\;\frac{1}{2}$), and ($\frac{1}{2}\;\frac{1}{2}\;0$) present in both but weaker in the needlelike crystal. More details are given in Section SI of the Supplemental Material (SM). Temperature-dependent measurements confirmed that the modulation was commensurate with the lattice and present over the entire measured temperature range (120–300 K). The additional vectors point to a larger face-centered lattice, but an attempted refinement with such a structure did not improve on that obtained with the expected Pnma unit cell. The nature of this modulation will require further exploration and is beyond the scope of this work; for now we note that WP has the most distorted structure (based on comparison to “ideal” orthorhombic lattice parameters) of any MnP-type binary [10]. This is a result of the large area associated with the 5*d* W orbitals [13], leading to more overlap with nearby atoms than in other MnP-type materials, and may have some bearing on the periodic lattice modulation.

**Figure 2:**
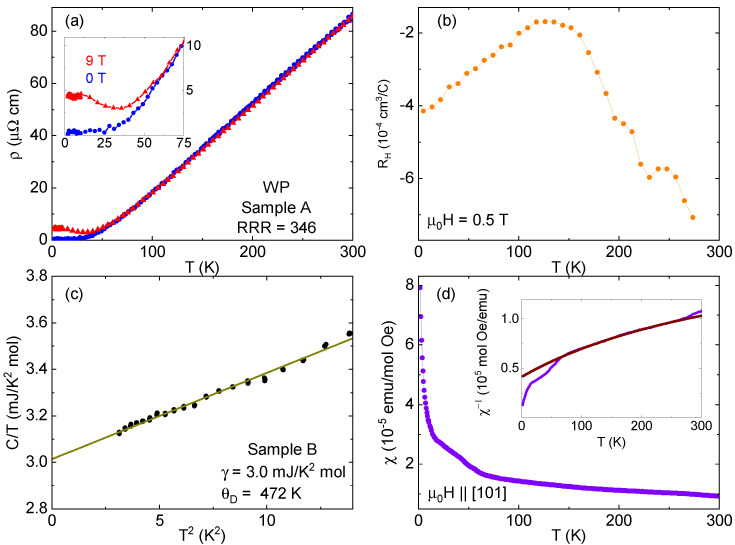
(**a**) Resistivity as a function of temperature for a WP single crystal with current along the *b*-axis in zero field and with a 9 T applied field. (**b**) The Hall coefficient (based on temperature sweeps in ±14 T) for a WP single crystal. (**c**) Low-temperature specific heat of a different WP single crystal. The green line is a fit to the Debye model, with extracted parameters noted. (**d**) Magnetic susceptibility of WP powder as a function of temperature. Data shown are field cooled, but there was no difference with zero-field cooling. Inset: An inverse susceptibility plot of the same data with a Curie–Weiss fit (maroon line) over the data from 100 to 250 K (see text for details). For (**a**,**b**,**d**), the field was applied parallel to [101].

The resistivity has a linear dependence at high temperature, before leveling off below 30 K [Figure 2a]. This is much like what was previously seen [12]; however, crystals grown with the ${\mathrm{WO}}_{3}$ method have substantially lower residual resistivity than those grown from WP polycrystals. The residual resistivity ratio (RRR, defined as $\rho_{300K}/\rho_{1.8K}$) exceeds 300 in some cases, with resistivity ρ values down to about 0.2 μΩ cm at base temperature. These are much higher and lower than the initial report on these materials (about 40 and 1 μΩ cm, respectively, as the previous work reported a 300 K resistivity that is about half of our value), and we believe they are a direct result of the different growth technique, where the materials are better able to mix in the gas phase. The lowest residual resistivities of other MnP-type phosphides are also about 0.1–0.3 μΩ cm [11,22,30], indicating that our growth technique approaches what may be a rough lower limit of resistivity in this family. Magnetoresistance becomes appreciable around 50 K, and dρ/dT is negative below 35 K in an applied field of 9 T. This is about the same “turn-on” temperature at which MR becomes significant in isostructural CrP [22] and FeP [11], as well as topological materials such as ${\mathrm{WTe}}_{2}$ [31].

In other materials, the minimum seen in ρ(*T*) when a field is applied is a sign of a change in carrier concentration or mobility ratios of carriers of different signs [11,31,32]. This is often reflected by a complicated temperature dependence of the Hall resistance. However, the large magnetoresistance of these samples at low temperatures makes it difficult to isolate a fully antisymmetric Hall effect signal. Instead, temperature sweeps were made at ±14 T with **H** ‖ [101], with the difference between the two field extremes used to calculate a slope, which implicitly assumes a linear Hall resistance [Figure 2b] but is acceptable for qualitative analysis. Conduction is electron-dominated at all temperatures; band structure calculations anticipate hole carriers as well, though they are expected to be from two-dimensional sheets at the Fermi surface [10]. There is a clear temperature dependence of the Hall coefficient $R_{H}$, including a broad maximum at 130 K. There is no clear feature below 50 K, in the vicinity of the increase in MR. Still, the nonmonotonic temperature dependence is similar to what has been seen in other materials with this structure [11,33,34].

The low-temperature specific heat data [Figure 2c] can be fit reasonably well by the Debye low-temperature model $C_{p}/T=\gamma +\beta T^{2}$. The extracted γ value of $3.0\frac{\mathrm{mJ}}{{\mathrm{mol}K}^{2}}$ is twice that reported for the needlelike samples [12]. The Debye temperature calculated from the slope of the C/T vs. $T^{2}$ plot is 472 K. The magnetic susceptibility χ of 94 mg of WP powder taken from the hot end of a growth ampule (to ensure there was no $W_{8}P_{4}O_{32}$) was also measured [Figure 2d]. Attempts were made with single crystals, but the combination of a small moment and still relatively small mass made it difficult to detect a signal. The data shown are only for field cooling at 0.5 T, but zero-field cooling or a higher field gave similar results. The susceptibility increases with cooling, with noticeable kinks coming at about 60 K and 15 K. However, there is no indication of long-range order. A fit to the data using the Curie–Weiss formula $\chi =\chi_{0}+\frac{C}{T-\Theta_{\mathrm{CW}}}$ over the range 100–250 K [Figure 2d, inset] gives values of $5.2\;\times \;10^{-6}\frac{\mathrm{emu}}{\mathrm{mol}\mathrm{Oe}}$ for $\chi_{0}$, accounting for parasitic para- and diamagnetic contributions; $1.8\;\times \;10^{-3}\frac{\mathrm{emu}K}{\mathrm{mol}\mathrm{Oe}}$ for the Curie constant *C*; and −92 K for the Curie–Weiss temperature $\Theta_{\mathrm{CW}}$, indicating dominant antiferromagnetic fluctuations.

**Figure 3:**
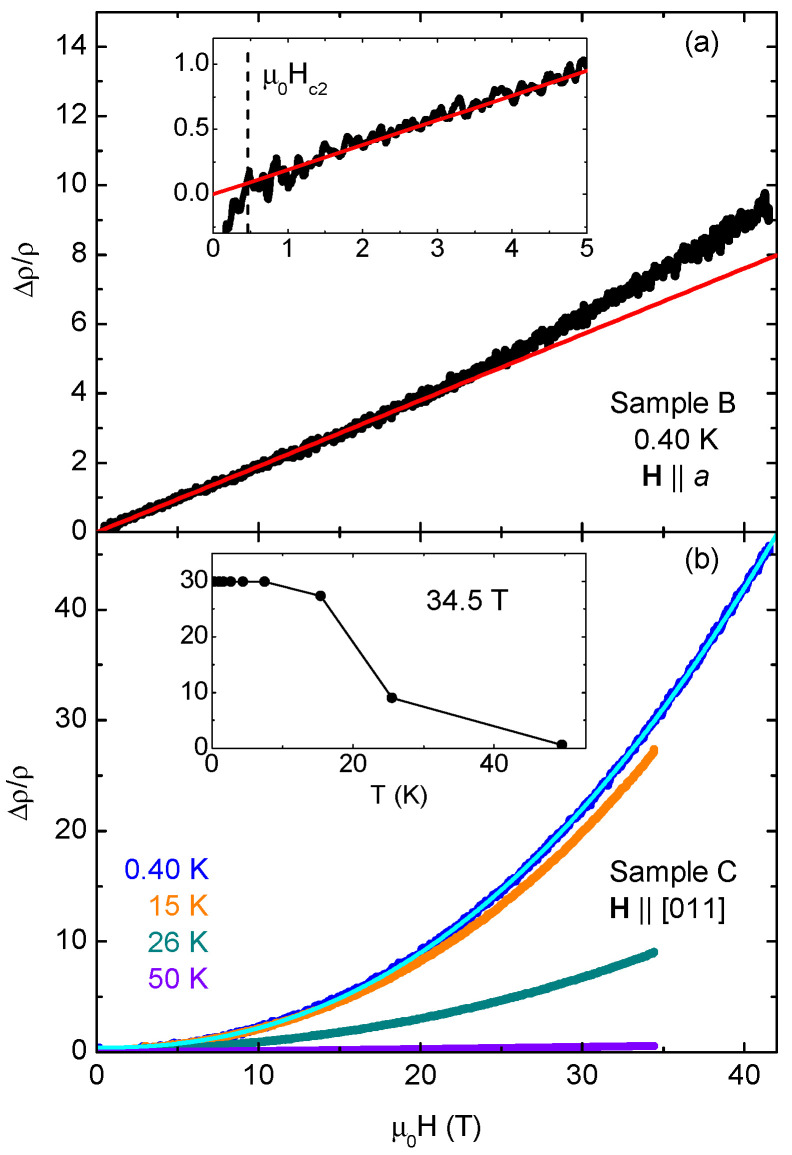
(**a**) Magnetoresistance (defined as $\frac{\rho \left(B\right)-\rho_{0}}{\rho_{0}}$, where $\rho_{0}$ is the lowest resistance above the superconducting transition) of a WP sample with **H** ‖ *a*-axis at 0.40 K up to 41.5 T. Data up to 3 K were practically indistinguishable, aside from superconducting transitions visible at a very low field in the data below 1.3 K. The red line is a linear fit to normal-state data from $H_{c2}$ (determined by the change in slope; see inset) to 20 T. (**b**) MR of a different WP crystal with **H** ‖ [011] at multiple temperatures. Data showed little variation from base temperature to 8 K, and the next lowest temperature was 15.4 K. The light-blue line is a power-law fit of the base temperature data, yielding *n* = 2.27. The inset shows MR at 34.5 T (the maximum field of the higher temperature measurements) as a function of temperature, including temperatures not shown in the main plot.

Two WP samples were measured at high fields at the NHMFL. One was only measured at very low temperatures with a field along the *a*-axis [Figure 3a]. It showed a nearly linear dependence on the field before gradually starting to curve upward above 20 T (a red line marks the 0–20 T fit). Data were nearly identical up to 3 K, the highest measured temperature, with no noticeable decrease in MR at the highest field. The increasing width of the signal at a high field is due to noise—no coherent quantum oscillations were found. The second sample had its [011] axis aligned with the field, and data were taken over a wider temperature range, allowing the drop in MR with increasing temperature to be seen [Figure 3b]. A generic power-law fit to the lowest-temperature data yields *n* = 2.27, with little deviation over the entire field range. The MR is also about five times larger at the base temperature and maximum field for this orientation than for the other angle.

Though we measured two quite different magnetoresistance forms and magnitudes of MR at a high field, we cannot make a comment about MR anisotropy since the data come from separate samples. However, the linearity of Sample B in Figure 3a up to 20 T is reminiscent of the high-field linear magnetoresistance seen in FeP [11] and (under pressure) CrAs [9], also starting from a very low field. There, it is attributed to “semi-Dirac points” in the band structure, points from which the dispersion is linear in a single-crystal direction. This linear dispersion in turn can give rise to linear, nonsaturating magnetoresistance when aligned with an applied field. While, for those two, linearity occurred at **H** ‖ *c*, in the absence of magnetic order, the same semi-Dirac point will appear at other places in the band structure and be crystallographically protected [10,11]. This means that a paramagnetic material such as WP could have linear magnetoresistance in other alignments.

### 3.2. Superconducting Properties

**Figure 4:**
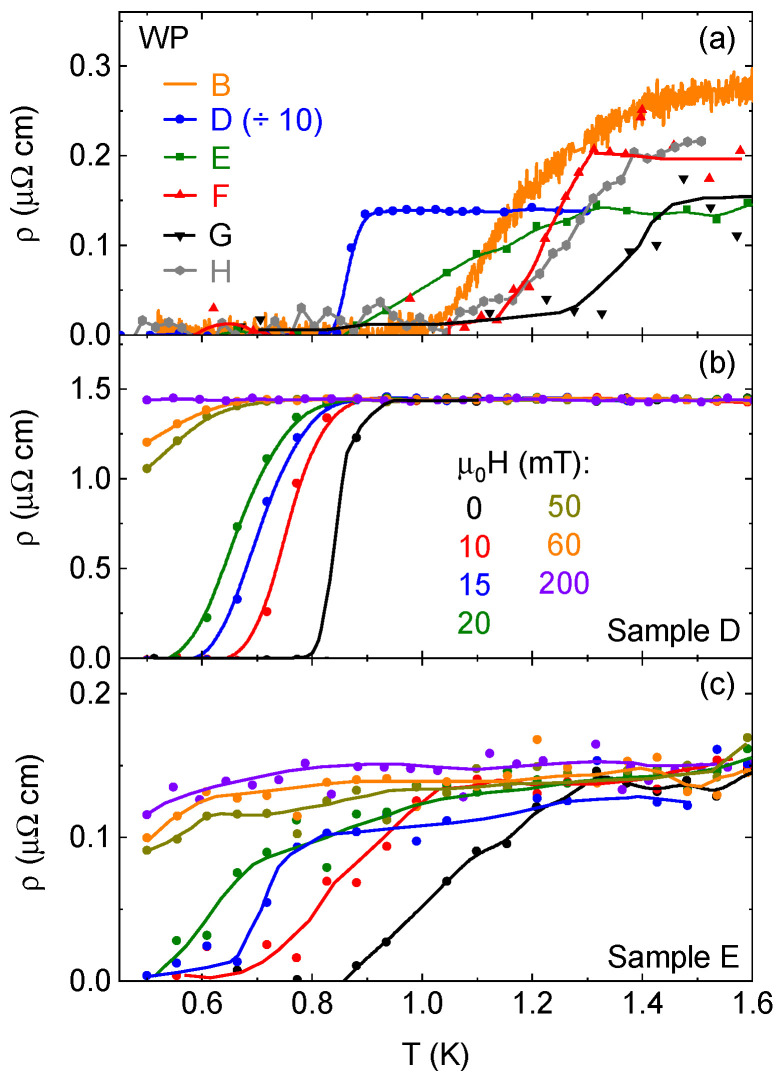
(**a**) Zero-field temperature sweeps, showing superconducting transitions, of five different WP single crystals. Sample D has had its resistivity reduced by a factor of 10 to fit the plot scale, while for Sample B, measured at the NHMFL, resistance was not converted to resistivity, so the units are arbitrary. The lower two panels are temperature sweeps in various fields for WP samples (**b**) D and (**c**) E, which were measured simultaneously. All lines are guides to the eye.

Figure 4a shows zero-field temperature sweeps for five WP samples, whose superconducting transitions vary in character. Sample D had a higher residual resistivity (1.5 μΩ cm, reduced by a factor of 10 in the figure) and more needlelike shape, despite being grown with the oxide method. Its superconducting transition at 0.85 K is very sharp, very similar to the first report of superconductivity in this material [12]. Four samples with higher RRR values, B and E-G, showed transitions that began and ended above 0.85 K, though they were also much broader. As shown in Figure S2 of the Supplemental Material, this may be a result of Ohmic heating resulting from using high currents to reduce noise. Sample B is the **H** ‖ *a*-axis sample from Figure 3a, and the data come from the zero-field cooldown at the NHMFL. The resistance values for that sample were not converted from resistance to resistivity, so the resistance has simply been scaled to fit to the plot. The data for samples B and E–H are noisier because the resistance is lower, due to both the lower residual resistivity and the different typical geometric factor of platelike samples. The contrast between the two morphologies can be seen in Figure 1b. While the platelike samples could be polished to try to maximize resistance, this could not be achieved to the extreme degrees of needlelike samples, which could have an as-grown length over 1 mm and a thickness of less than 50 μm. As a result, the platelike samples are closer to the noise floor of the measurement systems. This can influence the appearance of the resistive transition, as explored in further detail in Section SII of the SM. The elevated $T_{c}$ is, nevertheless, apparent. While affected by Ohmic heating from the applied current [SM, Figure S2], the broadness of the transitions seems to be an inherent feature of the samples. There is a clear contrast in transition width with the lower $T_{c}$ sample (D, Figure 4b), including for a sample measured at the same time, which would have had the same field environment (E, Figure 4c). In the high-field experiment, a superconducting transition was seen in field sweeps at temperatures up to 1.3 K. However, the ρ(B) data at very low field for such measurements were not reliable enough for further analysis.

**Figure 5:**
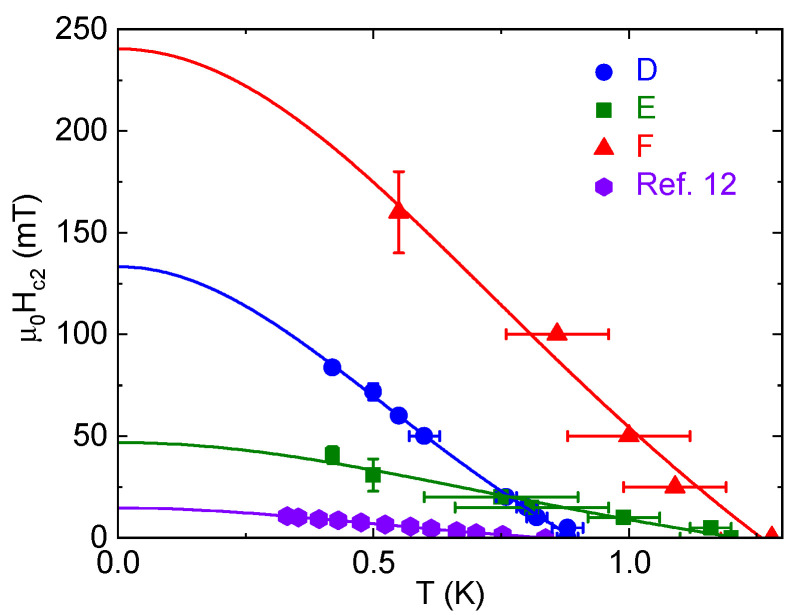
The critical field for the same WP samples shown in Figure 4a with matching colors and symbols, as well as the sample from the work of Liu et al. [12] with the highest critical field. The criteria for our samples was a 10% drop in resistivity; for the reference samples the transitions are sharp enough that different criteria would not impact the appearance of the data. Error bars mark uncertainty in either temperature (horizontal) or field (vertical) sweeps during measurements when the other variable was held constant. D and F have **H** ‖ *c*-axis, and for E and the reference sample, the orientations are unknown. Fits are made according to a Ginzburg–Landau formula, $H_{c2}\left(T\right)=H_{c2,0}\left[\frac{1-{\left(\frac{T}{T_{c}}\right)}^{2}}{1+{\left(\frac{T}{T_{c}}\right)}^{2}}\right]$.

The upper critical field $H_{c2}$ is, like $T_{c}$, increased in the platelike samples [Figure 5]. Given the broadness of the transitions, we took the beginning of the resistance drop (90% of normal-state resistivity) as $H_{c2}$, though some samples still had a slight positive slope in the normal state at low temperature, complicating this analysis [e.g., Figure 4c]. Error bars in Figure 5 come from an assessment of the noise in the signal. Samples G and H were not measured in a field. In comparing samples D and E, measured simultaneously, we see that the broadness of the higher-RRR samples is exaggerated by the field [Figure 4b]: the transition starts at higher temperature for E, but zero resistance occurs at about the same temperature for both. For two samples, D and F, both measured with **H** along the *c*-axis, we see that the higher $T_{c}$ results in a significantly higher critical field. Even so, in the case of sample D, $H_{c2}$ is roughly five times larger than the highest value reported by Liu et al. for samples with a similar $T_{c}$ and residual resistivity [12] and, in low-temperature field sweeps, is higher than that of sample E. This is not just a question of orientation, as rotational measurements on WP crystals grown from polycrystals have shown a change of only about a factor of two with angle [12,13]. The $H_{c2}$ vs. $T_{c}$ curves fit well (though less so for sample F) to the Ginzburg–Landau formula of the form $H_{c2}\left(T\right)=H_{c2,0}\left[\frac{1-{\left(\frac{T}{T_{c}}\right)}^{2}}{1+{\left(\frac{T}{T_{c}}\right)}^{2}}\right]$. That being said, our data points are primarily in the higher-temperature, more linear portion of the curve.

## 4. Discussion

Variation in $T_{c}$ for materials grown by CVT has precedent, for example, in the case of triplet superconductor ${\mathrm{UTe}}_{2}$. There, variations in growth conditions can change not only $T_{c}$ but also the RRR and the appearance of the heat capacity transition [35,36,37]. WP was predicted to be an unconventional topological crystalline superconductor [10]. The same is expected for CrAs and (upon suitable doping) MnP, the two isostructural compounds that superconduct under pressure with suppression of magnetic order [6,7,8]. The competition those two exhibit between magnetic order and superconductivity produces a phase diagram reminiscent of that seen in quantum critical materials [38], and in the case of CrAs, there is some experimental evidence for triplet superconductivity [39,40]. A non-*s*-wave gap could account for the $T_{c}$ variation in WP in samples with lower residual resistivity. When the superconducting gap is anisotropic, Cooper pairs can be more easily destroyed by scattering off areas where the gap is very small or zero. Samples with reduced scattering should have higher-temperature transitions, as in the case of multicomponent superconductor ${\mathrm{Sr}}_{2}{\mathrm{RuO}}_{4}$ [14,41,42].

The transitions are broader in the higher-RRR samples. As explored further in the Supplemental Material, this may be a result of using higher current values to reduce noise [Figure S2]. Though even when lower current was used, transitions did not appear as sharp as samples with a 0.8 K $T_{c}$. One possibility is filamentary superconductivity, which could be assessed with more bulk probes. However, while we attempted to measure the superconducting transition in specific heat and with a tunnel diode oscillator technique, in both cases there was no signal of superconductivity at any temperature, likely due to a low sample mass. Measurements that could confirm bulk superconductivity in oxide-grown crystals are essential for further study. In the absence of that, we have to look elsewhere to determine whether the $T_{c}$ variation is inherent to WP. The XRD measurements found no evidence of any other phases or nonstoichiometry that might produce a spurious, local zero-resistance signal. Additionally, the upper critical fields of the higher-$T_{c}$ samples are much larger than those found in the previous study [12]. Theoretical calculations for the $T_{c}$ of WP closely matched the previously observed value of about 0.84 K [29]. However, our values of γ and $\theta_{D}$ are roughly double those in the previous experiment and theory (about 1.3 mJ/mol $K^{2}$ and 240 K, respectively), which, in the absence of other changes, would raise $T_{c}$. A Bloch–Grüneisen fit of our resistivity data produces a value of the electron–phonon coupling constant $\lambda_{ep}$ that is very close to that obtained in the previous work [SM, Figure S3]. However, if superconductivity is indeed unconventional, it is questionable how much of a role electron–phonon coupling plays in determining $T_{c}$.

There is precedent for a higher temperature but broader transition in strongly correlated superconductors. In ${\mathrm{CeRhIn}}_{5}$, the resistive transition starts well above the temperature determined by heat capacity and is much broader for current in the ab plane compared to along the *c*-axis [15], even though the former has an extrapolated normal-state residual resistivity about an order of magnitude lower. This is attributed to a change in the antiferromagnetic state slightly above bulk $T_{c}$ and only appears for higher-quality samples. Multigap superconductors ${\mathrm{LaNiC}}_{2}$ [16] and SnSb [17] both have a difference between bulk and resistive $T_{c}$, with the zero-resistance state also having a much higher critical field. SnSb in particular is an interesting case, as it features a superstructure on top of the basic rock salt crystal symmetry, similar to the modulated structure seen in WP. This modulated structure could affect the orbital overlap and electronic configuration slightly so that a transition is visible above the typical superconducting temperature. Band hybridization, spin–orbit coupling, and an anisotropic gap, all expected to be relevant to a proper description of WP [10], could likewise be sensitive to slight variations. While the two samples on which single-crystal XRD were performed had nearly identical lattice parameters and both showed the modulation, it was stronger in the three-dimensional, higher-$T_{c}$ one.

This last point returns to the connection between growth method, morphology, crystal quality, and superconductivity. Oxide-assisted growth favors more 3D samples than WP-powder-based synthesis, and we only find higher-temperature resistive transitions in the 3D samples. XRD has shown no difference in the *Pnma* lattice parameters between the two morphologies. The structural differences between the samples are then the modulation strength and the reduced impurity concentration, as indicated by residual resistivity. How directly the two could be linked is unclear. The relation between the possible multiple superconducting phases and the predicted topological crystalline superconductivity is, likewise, something to be explored.

## 5. Conclusions

Vapor transport growth using ${\mathrm{WO}}_{3}$ and elemental P has been found to produce high-quality WP single crystals, with low-temperature scattering near the lower limit of the MnP family. Oxide-assisted samples also have a higher temperature but a broader resistive superconducting transition, with a higher critical field. This variation could emerge from the predicted nontrivial topological superconductivity of WP, or the modulated crystal superstructure. Deeper investigation will help sort out the nature of this superconducting phase and its relationship with predicted topological properties, as well as the electronic and crystal structure of WP.

## Figures and Tables

**Table 1:** Atomic position and anisotropic displacement parameters (in units of ${Å}^{2}$) for a WP single crystal at 120 K. $U_{12}$ and $U_{23}$ are both zero.

Atom	x	y	z	$U_{eq}$	$U_{11}$	$U_{22}$	$U_{33}$	$U_{13}$
W	0.51323(3)	0.25	0.68851(3)	0.00125(8)	0.0074(10)	0.00195(10)	0.00106(10)	−0.0006(4)
P	0.18441(17)	0.25	0.43383(18)	0.00205(18)	0.0022(4)	0.0021(4)	0.0019(4)	−0.0004(3)

## Data Availability

The original contributions presented in this study are included in the article/Supplementary Materials. Further inquiries can be directed to the corresponding author.

## Supplementary Materials

The following supporting information can be downloaded at https://www.mdpi.com/article/10.3390/ma1010000/s1.
