# Supplementary material for: Enhancement of Superconductivity in WP via Oxide-Assisted Chemical Vapor Transport
Source: Materials (Basel). 2025 Sep 29;18(19):4529. doi: 10.3390/ma18194529 (PMC12525612; doi:10.3390/ma18194529)
Supplement: Supplementary file 1 [file materials-18-04529-s001.zip › materials-3881661-supplementary.pdf]

Supplemental Material: Enhancement of Superconductivity in WP via Oxide-Assisted Chemical Vapor Transport

Daniel J. Campbell,[1] Wen-Chen Lin,[1] John Collini,[1] Yun Suk Eo,[1] Yash Anand,[1]
Shanta Saha,[1] David Graf,[2] Peter Y. Zavalij,[3] and Johnpierre Paglione[1,4,*]
[1]*Maryland Quantum Materials Center, Department of Physics, University of Maryland, College Park, Maryland 20742, USA*
[2]*National High Magnetic Field Laboratory, 1800 East Paul Dirac Drive, Tallahassee, Florida 32310, USA*
[3]*Department of Chemistry, University of Maryland, College Park, Maryland 20742, USA*
[4]*Canadian Institute for Advanced Research, Toronto, Ontario M5G 1Z8, Canada*
(Dated: September 19, 2025)

I. CRYSTAL STRUCTURE MODULATION

As noted in the main text, a crystal structure modulation was observed in both samples of WP on which single crystal diffraction measurements were performed, one more three dimensional and one needlelike. Figure S1 shows images from the more 3D crystal, for which the modulated structure had a stronger intensity, at $T = 150$ K. There are in fact several coexisting long range modulation vectors, as demonstrated in Fig. S1. Panel (a) shows the $b - c$ plane in reciprocal space, where the Bragg peaks can be identified as the dark spots surrounded by slight shading. The weaker intensity spots in between come from the modulation vectors: the $[\frac{1}{2}\ \frac{1}{2}\ 0]$ (and cyclic permutations thereof) modulation extending vertically and horizontally, and the $[0\ \frac{1}{7}\ \frac{1}{7}]$ modulation diagonally. The lower two panels highlight two different planes ([100]-[011] and [100]-[001]), where one or both of the modulated vectors can again be seen.

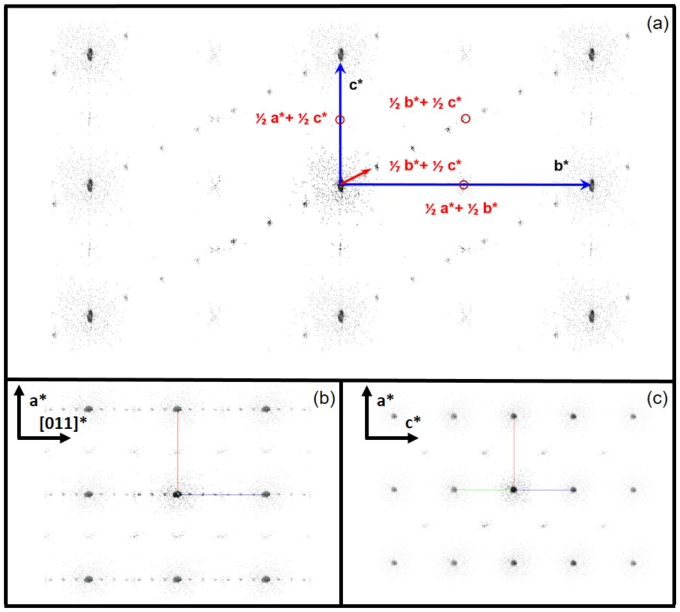

FIG. S1. (a) The reciprocal space reflections from single crystal diffraction of a WP sample in the $b - c$ plane. The lower intensity spots correspond to the multiple crystal structure modulations, which are labeled. (b) The [100] (vertical) and [011] (horizontal) plane projection, where the $[0\ \frac{1}{7}\ \frac{1}{7}]$, $[\frac{1}{2}\ \frac{1}{2}\ 0]$, $[0\ \frac{1}{2}\ \frac{1}{2}]$, and $[\frac{1}{2}\ 0\ \frac{1}{2}]$ modulations are all still visible. (c) A similar view of the ac plane, where modulation reflections are visible along the diagonals. Data in the figure were taken at 150 K, though modulation was seen in all measurements 120-300 K.

* paglione@umd.edu

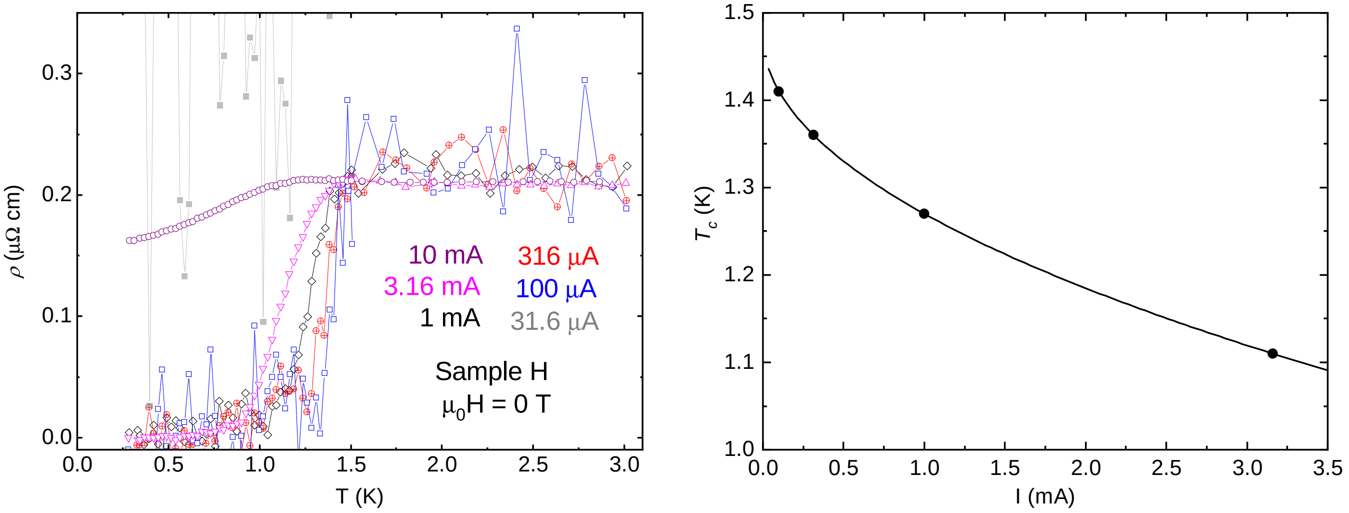

FIG. S2. (a) Measurements of WP crystal H during the same experiment at various currents, showing the dependence of the noise (due to the small voltage) and transition temperature (due to Ohmic heating) on applied current. For this sample, 0.2 μΩ cm corresponds to about 10 μΩ. (b) Dependence of T_c (defined here as 50% of the normal state resistance) on current, with a power law fit as a guide to the eye.

II. MEASURING T_c IN SAMPLES WITH A SMALL RESISTANCE

As seen in Fig. 4 of the main text, the resistivity of oxide-grown WP samples just above the superconducting transition approaches 100 nΩ cm. The crystals' three dimensional character means extensive polishing to small dimensions is required to maximize the geometric factor and thus the resistance. However, such extreme differences in physical dimensions risk breaking the sample and are generally difficult. Therefore, our data still show a noticeable effect from that noise that, as noted in the main text, makes exact determination of quantities like T_c and H_{c2} difficult.

The measured voltage can be increased to reduce the noise level by increasing the applied current, but at low temperatures the effect of Ohmic heating is significant. This is demonstrated by Fig. S2(a), which shows measurements on the same sample at various currents. It can be seen that as the current increases, the noise is reduced due to the higher voltage, but T_c also decreases because the sample is now warmer than the temperature measured by the thermometer, and the transition becomes broader. The normal state resistivity becomes noisier, but its average value shows little change because it is constant at low temperatures. For this and all samples, the curves exhibited in the main text had to balance an acceptable noise level and minimal sample heating. This in fact means that our $T_{c,onset}$ values may underestimate what would be measured for $I \rightarrow 0$. For Sample H we used the 1 mA data. We note that even with a 10 mA current the beginning of a transition is seen about 1 K, though it is incomplete down to 250 mK. Data with higher applied current also overestimate the transition width, though even at 100 μA the transition appears wider than for the lower T_c sample at a higher current. Our main point still stands, as we have demonstrated that with oxide-assisted growth T_c can be enhanced over the previously reported 0.8 K value.

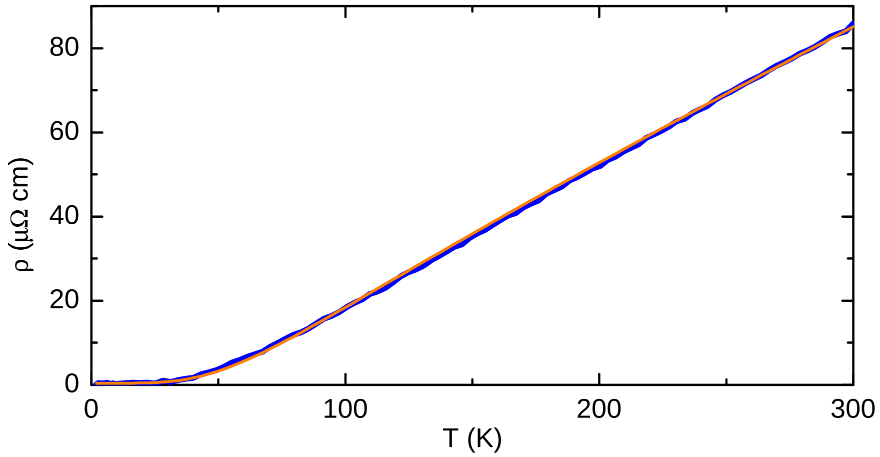

FIG. S3. The same 0 T resistivity data shown in Fig. 2(a) of the main text (blue), fit to the fifth-order Bloch-Grüneisen formula (Eq. 1, orange). The resistivity at 1.8 K was subtracted before fitting so that $\rho = 0$ μΩ cm at low temperature.

III. BLOCH-GRÜNEISEN FITTING OF THE RESISTIVITY

As was done in previous work on WP [1], we can fit resistivity data very well with the fifth-order Bloch-Grüneisen formula:

$$\rho(T) = \rho_0 + A\left(\frac{T}{\theta_{D,\rho}}\right)^5 \int_0^{\theta_{D,\rho}/T} \frac{x^5}{(e^x-1)(1-e^{-x})}, dx \quad (1)$$

The results for the same resistivity data as in Fig. 2(a) of the main text are shown in Fig. S3 (note that the residual resistivity ρ_0 was subtracted before fitting, leaving one term on the right hand side of Eq. 1). The extracted Debye temperature $\theta_{D,\rho}$ is 317 K. This differs from the values obtained in the previous paper (238 K) and from our own specific heat data ($\theta_{D,\beta} = 472$ K). Following the previous work, we can insert this value into the McMillan formula, written in a form so as to estimate the electron-phonon coupling strength λ_{ep}:

$$\lambda_{ep} = \frac{1.04 + \mu^* \ln\left(\frac{\theta_D}{1.45T_c}\right)}{(1-0.62\mu^*)\ln\left(\frac{\theta_D}{1.45T_c}\right) - 1.04} \quad (2)$$

Using the same estimate of $\mu^* = 0.13$, and roughly estimating the higher T_c achieved in our growth method (which, as shown, is sample dependent and based on broader transitions) as 1.2 K, we calculate a value $\lambda_{ep} = 0.458$. This is quite similar to the value of 0.453 obtained with the Debye and superconducting temperatures of the previous group, as the logarithmic dependence of λ_{ep} on θ_D/T_c weakens the effect of the roughly 12% decrease from that study to ours. In fact, even using our much higher $\theta_{D,\beta}$ we get a value of 0.430, still close to the other two.

On one hand, the similarity of these values shows that different T_c in our samples could be reasonably expected from the variation in θ_D. However, the McMillan formula has a limited applicability, by its nature assuming an electron-phonon Cooper pairing mechanism, and even then is not accurate for all phonon density of states spectra [2]. Given the predictions of unconventional superconductivity in WP and Raman effect measurements indicating the electron-phonon coupling alone cannot account for superconductivity [3], it is probable that Eq. 2 cannot be applied in such a straightforward way. Electron-phonon coupling has been found to play a role, albeit not necessarily the central one, in unconventional superconductors such as the cuprates [4, 5]. The link between increases in θ_D and T_c may be relevant, but likely not in a way accurately captured by this formalism.

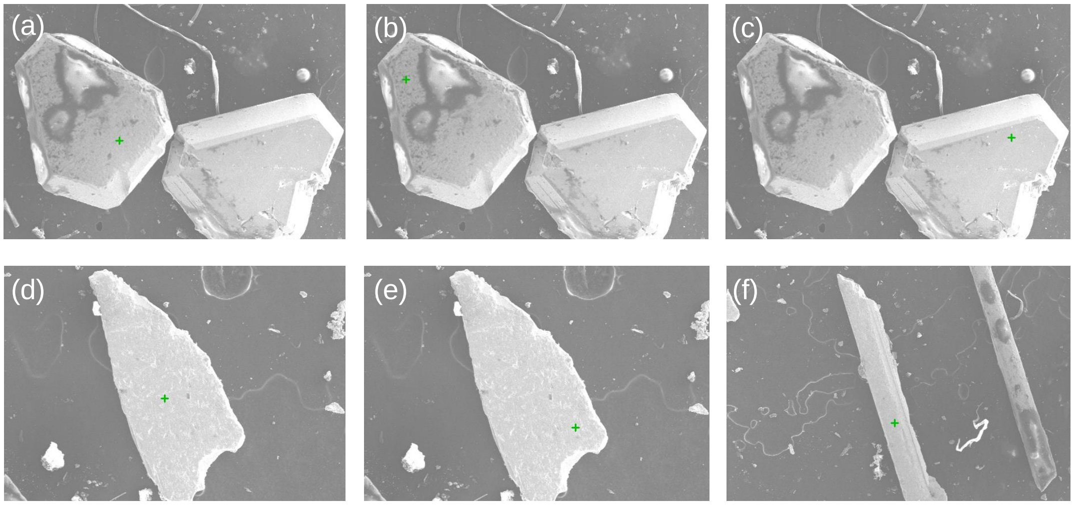

FIG. S4. Scanning electron microscope images of as-grown WP samples. Green crosshairs indicate the points were energy dispersive x-ray spectroscopy (EDS) measurements were made

IV. EDS RESULTS

We performed energy-dispersive x-ray spectroscopy (EDS) on a number of samples to assess composition. Figure S4 shows some representative results, indicating that samples consistently have quite close to a 1:1 ratio of W and P. That being said, the W concentration always seems to be slightly higher than P. We noticed the same phenomenon in synthesizing FeP [6], and speculate that it may be due to the high vapor pressure of P, leading to a slightly higher vacancy rate during crystal formation.

TABLE S-I. Atomic percentages of W and P at the points sampled in each panel of Fig. S4.

Spot	W (%)	P (%)
a	51.2	48.8
b	50.3	48.7
c	51.9	48.3
d	53.5	46.5
e	51.5	48.5
f	51.1	48.9

[1] Z. Liu, W. Wu, Z. Zhao, H. Zhao, J. Cui, P. Shan, J. Zhang, C. Yang, P. Sun, Y. Wei, S. Li, J. Zhao, Y. Sui, J. Cheng, L. Lu, J. Luo, and G. Liu, Superconductivity in WP single crystals, Phys. Rev. B **99**, 184509 (2019).
[2] C. K. Poole, H. A. Farach, and R. J. Creswick, *Handbook of Superconductivity* (Elsevier, 1999).
[3] Y. Zhang, L. Yan, W. Wu, G. He, J. Zhang, Z. Ni, X. Jiang, M. Qin, F. Jin, J. Yuan, B. Zhu, Q. Chen, L. Zhou, Y. Li, J. Luo, and K. Jin, Single-crystalline transition metal phosphide superconductor WP studied by Raman spectroscopy and first-principles calculations, Phys. Rev. B **105**, 174511 (2022).
[4] A. Lanzara, P. Bogdanov, X. Zhou, S. Kellar, D. Feng, E. Lu, T. Yoshida, H. Eisaki, A. Fujimori, K. Kishio, *et al.*, Evidence for ubiquitous strong electron–phonon coupling in high-temperature superconductors, Nature **412**, 510 (2001).
[5] D. Reznik, L. Pintschovius, M. Ito, S. Iikubo, M. Sato, H. Goka, M. Fujita, K. Yamada, G. Gu, and J. Tranquada, Electron–phonon coupling reflecting dynamic charge inhomogeneity in copper oxide superconductors, Nature **440**, 1170 (2006).
[6] D. Campbell, J. Collini, J. Sławińska, C. Autieri, L. Wang, K. Wang, B. Wilfong, Y. Eo, P. Neves, D. Graf, *et al.*, Topologically driven linear magnetoresistance in helimagnetic fep, npj Quantum Mater. **6**, 38 (2021).
